# Metal Ions Induce Liquid Condensate Formation by the F Domain of *Aedes aegypti* Ecdysteroid Receptor. New Perspectives of Nuclear Receptor Studies

**DOI:** 10.3390/cells10030571

**Published:** 2021-03-05

**Authors:** Anna Więch, Aneta Tarczewska, Andrzej Ożyhar, Marek Orłowski

**Affiliations:** Laboratory of Biochemistry and Molecular Biology, Department of Biochemistry, Molecular Biology and Biotechnology, Faculty of Chemistry, Wrocław University of Science and Technology, Wybrzeże Wyspiańskiego 27, 50-370 Wrocław, Poland; anna.wiech@pwr.edu.pl (A.W.); aneta.tarczewska@pwr.edu.pl (A.T.); andrzej.ozyhar@pwr.edu.pl (A.O.)

**Keywords:** intrinsically disordered proteins, intrinsically disordered regions, nuclear receptors, *Aedes aegypti*, F domain, EcR, liquid-liquid phase separation, Cu^2+^-induced LLPS

## Abstract

The superfamily of nuclear receptors (NRs), composed of ligand-activated transcription factors, is responsible for gene expression as a reaction to physiological and environmental changes. Transcriptional machinery may require phase separation to fulfil its role. Although NRs have a similar canonical structure, their C-terminal domains (F domains) are considered the least conserved and known regions. This article focuses on the peculiar molecular properties of the intrinsically disordered F domain of the ecdysteroid receptor from the *Aedes aegypti* mosquito (AaFEcR), the vector of the world’s most devastating human diseases such as dengue and Zika. The His-Pro-rich segment of AaFEcR was recently shown to form the unique poly-proline helix II (PPII) in the presence of Cu^2+^. Here, using widefield microscopy of fluorescently labeled AaFEcR, Zn^2+^- and Cu^2+^-induced liquid-liquid phase separation (LLPS) was observed for the first time for the members of NRs. The perspectives of this finding on future research on the F domain are discussed, especially in relation to other NR members.

## 1. Characteristics of NRs

**Figure 1:**
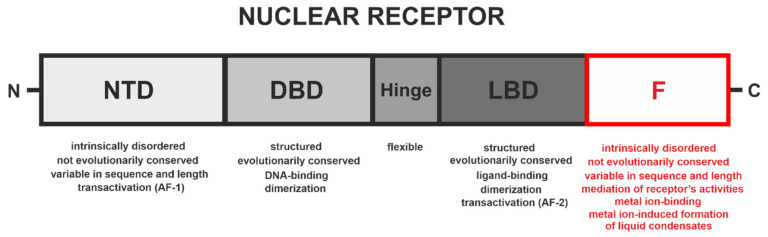
Scheme of the overall structural and functional organization of nuclear receptors (NRs). Nuclear receptors contain at least four distinct domains: a N-terminal domain (NTD), a DNA-binding domain (DBD), a hinge region, and a ligand-binding domain (LBD). Some NRs possess an additional C-terminal F domain. The contribution of each domain to the NR’s activities is different. The most important functions and properties located in the individual domains are listed below the scheme [1]. The F domain is marked in red [10,11].

Nuclear receptors (NRs) are a specific superfamily of the most abundant class of ligand-activated transcription factors. These transcription factors play a critical role in the modulation of the expression of target genes by selective binding to appropriate genomic DNA response elements. They are responsible for the regulation of fundamental biological processes such as development, tissue and metabolic homeostasis, cell proliferation, metamorphosis, and reproduction in animals [1,2]. The activities of most NRs are regulated endogenously by small lipophilic ligands such as retinoids, steroids, thyroid hormones, or dietary lipids [3]. NRs are also the targets of other cellular signaling pathways that modify post-translationally receptors, or their co-modulators, in turn affecting their activities and functionality [4]. Their remarkable properties of transducing extracellular signals via the binding of specific ligands to directly modulate gene expression make them critical pharmaceutical and therapeutic drug targets [5]. Disrupted NR signaling pathways contribute to numerous diseases such as cancer or diabetes [5,6,7]. Although diversity in the overall shape, size, and plurality of specific ligands can be observed, the majority of the known members of NRs share a common modular structure (Figure 1) that generally consists of four domains [8,9].

The N-terminal domain (NTD) is responsible for ligand-independent transactivation (AF-1). The most characterized NTDs to date exhibit features of intrinsically disordered proteins (IDPs) [12,13,14,15,16,17,18,19]. Such a property is advantageous for their inter- and intramolecular interactions [20], as NTDs can undergo disorder-to-order (or partially order) transitions as a result of co-activator/co-repressor binding, interaction with DNA or posttranslational modifications (PTMs) [21,22,23]. An NTD is followed by a highly conserved DNA-binding domain (DBD). Next, the disordered and flexible hinge region serves as a linker between the DBD and the evolutionarily conserved ligand-binding domain (LBD), which is responsible for the ligand molecule binding and ligand-dependent activation of transcription (AF-2), but also mediates homo- or heterodimerization with another NR superfamily member. Some NRs own an additional hypervariable (in terms of length and sequence) C-terminal F domain (Figure 1) [1,3,10,11,24]. The C-terminal F domains of some NRs are not clearly defined. Amino acid residues, following the last helix (helix 12) of LBDs, define the F domains, and due to their short sequence, in many mammalian NRs, they are often considered as a part of the LBDs.

## 2. Transcription Factors in the Cellular Response to Mosquito-Borne Infections

Tropical and subtropical areas stand out in terms of their lush vegetation, various animal species, and changing weather conditions. Most of the world’s population live in these areas and struggle with the health problems that frequently occur there. Environmental conditions are favorable for insect reproduction, and therefore for insect-dependent disease transmission.

Caused by one of the 4 serotypes of the dengue virus, dengue is one of the greatest threats in the world. Annually, about 390 million people get infected, with 96 million of them developing symptoms [24,25]. Most people suffer from a characteristic rash, headache, and joint and muscle pain. Dengue fever may develop into much more severe states: dengue hemorrhagic fever (DHF) and dengue shock syndrome (DSS), which require immediate hospitalization and specific treatment [26]. Another pathogen, like dengue virus, belonging to the *Flaviviridae* family, is Zika virus—first reported in 1947 in Uganda [27], sequenced in 2007 [28]. Until its outbreak in 2015 in Brazil (more than 1.5 million infections), only several cases of Zika infection were reported [29]. Some patients undergo the disease with fever, rash, and joint and muscle pain, but most of them do not show any symptoms [30]. This is the actual problem, because Zika virus can be transmitted sexually or passed from the mother to the fetus. The mother’s infection may result in neurological disorders for the child, like microencephaly [31]. Yet another mosquito-borne disease is one of the deadliest on Earth-malaria. In 2018 alone, 228 million cases were reported [32]. After the blood meal, *Plasmodium falciparum* sporozoites (formed through fertilization) are transferred to human hepatocytes. Later, merozoites invade human erythrocytes, in turn causing erythocytic schizogony [33]. Shivers, fever, profuse sweating and headache are the first symptoms of malaria infection. Without proper treatment, patients may experience anemia, convulsions and coma [34].

### 2.1. Human Cellular Response to Viral Infection Involves Changes in Gene Expression

The regulation of gene expression requires the balanced orchestration of adjustable complexes of many specific transcription factors, modulators and signal transduction. Modulation of gene expression profiles by NRs is an allosteric process where binding ligands and specific DNA sequences initiate the recruitment of diverse transcription factors, co-activators, co-repressors and elements of the chromatin remodeling machinery to transactivate or transrepress the expression of target genes. Symptoms of dengue, Zika or malaria diseases are extensive and caused by a series of metabolic pathways. Some of them involve transcription factors to maintain the balanced orchestration of gene expression.

CREB3 (cyclic AMP-responsive element-binding protein 3), which enhances the transcription of genes involved in secretory pathways, takes part in the cellular response to Zika and dengue virus infections [35,36]. After infection with Zika virus, the host’s cells are forced to produce viral proteins, and therefore the secretory demands increase and cause endoplasmic reticulum stress. Transcriptome analyses of human cells infected with Zika virus, conducted by Moni et al., revealed upregulation of pathways concerning endoplasmic reticulum functioning [37]. To maintain endoplasmic reticulum homeostasis after infection with Zika virus, the expression level of the CREB3L2 gene (with other genes associated with this pathway) can be three times higher [37].

### 2.2. Transcription Factors Involved in the Mosquito’s Response to Pathogens

What is interesting is that the parasite itself is able to trigger a mechanism that will enable it to survive in the host’s cells [38,39,40]. The role of the transcription factors engaged in these mechanisms cannot be ignored. In *Anopheles*, the expression of antimicrobial peptides (AMPs), i.e., gambicin or attacin, is controlled by the Rel1 and Rel2 transcription factors. Their translocation to the nucleus depends on the activation of the Toll and Imd pathways, respectively [41]. In mosquitos, after ingestion, gametocytes turn into gametes, and after fertilization a zygote occurs. It develops into sporozoites, which are present in the salivary glands of mosquitos. Before transmitting *Plasmodium* to human, *A. gambiae* shows an immune response to the presence of the parasite. Disrupted parasite development is achieved by an increased level of reactive nitric oxide (NO). A high level of NO is caused by NO synthase (NOS), the transcription of which is mediated by STAT-A in *A. gambiae*. Interestingly, the mRNA level of STAT-A is controlled by its ortholog—STAT-B [41]. The so-called JAK-STAT mechanism has also been described as a part of the immune response of *A. aegypti* to dengue infection [42]. Most of the physiological effects described above are related to changes in the activity of transcription factors. It was recently shown that the development of *P. falciparum* parasites in *A. gambiae* females is linked to physiological processes controlled by 20-hydroxyecdysone (20E), which is a physiological ligand of the insect ecdysteroid receptor (EcR) during egg development [43]. The analysis of both eggs and parasite counts in individual females unveiled an unexpected positive correlation between mosquito and parasite fitness that may be dependent on 20E signaling [43]. However, the knowledge of the functional roles of NRs at the molecular level in such events in humans and insects is still obscure and limited.

## 3. Liquid-Liquid Phase Separation in Gene Expression

Recently, liquid-liquid phase separation (LLPS) emerged as a mechanism in which cells organize their interior, separate, and then segregate biochemical processes [44,45,46,47]. This thermodynamically driven process leads to the formation of barrier-free cellular bodies known as membraneless organelles (MLOs) [48,49]. MLOs are self-regulated liquid condensates, the components of which can diffuse freely and be exchanged rapidly with the surrounding milieu [45,50,51]. Interestingly, observations of molecule kinetics lead to the conclusion that although MLOs are composed of a number of different molecules [52], only a small fraction of them is needed to maintain the integrity of the condensates [50]. The majority of the MLOs’ components diffuse into the condensate due to interactions with scaffold molecules. The composition of these so-called client molecules changes, and therefore the functional properties of MLOs also change [50]. LLPS leads to the formation of MLOs in response to changes in the surrounding milieu [44]. Their formation may be triggered by stress factors [51] or be due to biochemical changes of molecules such as PTMs [53], the presence of ligands [54], and changes in the concentration of certain molecules [55]. The fact that pathogens utilize LLPS for invading host cells can also be seen to be interesting. For example, upon infection in the cytoplasm, viral ribonucleoprotein complexes alter the translation of cellular proteins by interfering with stress granules [56]. The intrinsically disordered regions (IDRs) of the EBNA2 and EBNALP transcription factors of the Epstein-Barr virus participate in nuclear LLPS in newly infected cells [57]. Following transcription of viral RNA, the N protein from the SARS-CoV-2 phase separates with RNA and forms liquid condensates which are a precursor for new virions [58,59]. This mechanism is also common for other types of viruses [60,61,62].

### Liquid-Liquid Phase Separation in Transcriptional Regulation by NRs

LLPS is important for the regulation of various aspects of gene expression, including transcription [63,64,65,66,67,68,69]. One of the first indications that transcription may depend on LLPS was provided by Hnisz et al., who developed a model describing the unique properties of macromolecular complexes formed on super-enhancers [65]. Sabari et al. showed that transcription co-activators condensate to super-enhancers and form liquid condensates [67]. Further research revealed that some transcription factors that possess IDRs can undergo spontaneous LLPS, in turn concentrating the transcriptional machinery and allowing the transcription to proceed [63]. This finding sheds new light on the mechanism by which transcription factors and transcription coactivators regulate gene expression. To date, little is known about the ability of NRs to induce LLPS. It was shown that the NTD of the androgen receptor (AR) can form liquid condensates in a concentration-dependent manner [70]. The AR is a substrate for Speckle-type POZ protein (SPOP) [71]. The interaction of AR and SPOP leads to proteasomal degradation of AR. Both proteins were shown to localize in liquid-like nuclear compartments. Mutations in SPOP, which alter its activity, are associated with some types of solid tumors in humans. It is important that the cancer-associated mutation disrupts the substrate-associated phase separation, in turn leading to an aberrant distribution of the protein between MLOs [70]. It has also been shown that the NTD of RXRγ can form liquid condensates in a temperature-dependent manner [19]. In fact, many proteins undergo phase separation in relation to changes of temperature [44,72]. One of the best known examples are proteins which form stress granules when the temperature decreases [51,73]. The NTD of RXRγ, however, undergoes a phase transition when heated above a critical temperature [19]. These properties were attributed to the fact that this domain is enriched in Pro and Gly residues encoded in Pro-Xn-Gly motifs (Xn residues are preferably nonpolar) with residues that are largely nonpolar [19,74]. The glucocorticoid receptor (GR) was also found to be distributed in the nucleus in a specific dotted pattern [75]. GR foci contain some transcription co-activators, including some subunits of the Mediator complex, which indicates that these condensates are an indispensable part of the transcription regulation machinery [76]. Similar observations were made for the estrogen receptor (ER) [63]. It is interesting that the AR and RXRγ induce LLPS by the NTD [19,70], whereas the GR and ER do not. In the case of GR removal of the C-terminal, the LBD drastically affected foci formation [76]. Likewise, the ER also forms liquid condensates when bounded in a ligand-dependent manner [63]. These examples show that NRs may influence gene expression using the LLPS to regulate their activity. However, due to insufficient evidence, the regions or domains responsible for provoking the process cannot be generalized. This is a new challenge in the study of NRs. At present, we also do not know if NRs in liquid condensates serve as scaffold molecules responsible for the formation of a condensate or as clients recruited to the preformed condensates similarly to client UBQLN2 recruited to stress granules [77]. In either case, NRs need to have specific features in their sequences which determine their ability to partition into liquid condensates. Their determination can contribute to a better understanding of the NRs’ functionality as transcription factors.

## 4. F Domains of Nuclear Receptors

**Figure 2:**
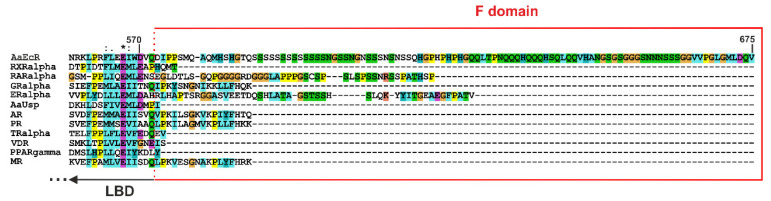
The F domains of various nuclear receptors are not evolutionarily conserved. The used abbreviations and the UniProt accession numbers (in parentheses) are as follows: *A. aegypti* EcR (AaEcR, GenBank: AAA87394.1), human retinoid X receptor α (RXRα, UniProt: P19793), human all-trans retinoic receptor (RARα, UniProt: P10276), human glucocorticoid receptor α (GRα, UniProt: P04150), human estrogen receptor α (ERα, UniProt: P03372), *A. aegypti* Usp protein (AaUsp, UniProt: Q9GSG7), human androgen receptor (AR, P10275), human progesterone receptor (PR, UniProt: P06401), human thyroid hormone receptor (TR, UniProt: P10827-2), human vitamin D receptor (VDR, UniProt: P11473), human peroxisome proliferator-activated receptor γ (PPARγ, UniProt: P37231), and human mineralocorticoid receptor (MR, UniProt: P08235). Residue numbering corresponds to the sequence of AaEcR (GenBank: AAA87394.1). Sequences corresponding to the F domain are boxed in red. All representative amino acid sequences were compared using the ClustalΩ tool [79].

When compared to the F domain of *A. aegypti* EcR (AaEcR) (ca. 107 aa) or the EcR from *D. melanogaster* (ca. 190 aa), the F domains of mammalian NRs are relatively short (from several to several dozen amino acid residues) [78]. As was mentioned above, the presence of F domains is not conserved in the NR superfamily, and the sequences of known F domains are highly variable in terms of length and sequence (for example see Figure 2). This remarkably variety is also visible among the members of the EcR family (Figure S1 in the Supplementary Materials). These short sequences are often considered as short extensions of the last helix in LBDs. Patel and Skafar extensively described the activities of the F domains of such NRs as ERα, ERβ, GR, PR, AR, MR, RXRα or RARα [78]. Although the F domains of the described NRs possess different lengths and they are not evolutionarily conserved in mammals (Figure 2 and Figure S2 in the Supplementary Materials), and the fact that only part of them seem to be intrinsically disordered (Figure S3 in the Supplementary Materials), they all appear to regulate the activities of receptors. They seem to affect dimerization, interactions with other proteins, and the stabilization of ligand binding, and thus contribute to the regulation of transcriptional activation [78].

Concerning the F domain role in the functioning of NRs, two papers during the last five years are worth paying attention to. Bianchetti and co-workers showed that the F domain of the glucocorticoid receptor-α (GRα) forms a steric hindrance to the well-known canonical LBD dimer assembly [80]. According to the authors, the currently accepted GRα homodimer structure and experimental investigations of the alternative architectures should be reexamined [80]. In the case of the F domain of the ERα, it was shown that this region governs the species-specific tamoxifen-mediated transcriptional activity of the ERα [81]. According to our current knowledge, none of the described F domains of NRs have the propensity to form metal ion-induced PPII [10] or undergo LLPS.

### 4.1. F Domain of the Ecdysteroid Receptor (EcR) from A. aegypti

**Figure 3:**
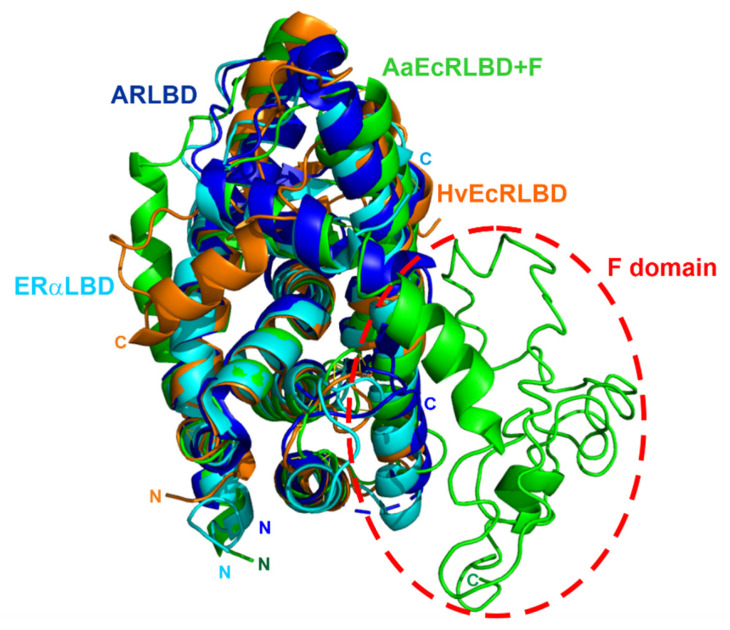
Superposition of the corresponding Cα atoms of the 3D structures of the LBDs of the exemplary representatives of NR LBDs with the 3D model of AaEcRLBD+F. The figure shows the superposition of the corresponding Cα atoms of ARLBD (blue) (PDB accession code 4oea), ERαLBD (cyan) (PDB accession code 1g50), EcRLBD from *Heliothis virescens* (HvEcRLBD) (orange) (PDB accession code 2r40) and the 3D model of AaEcRLBD with the F domain (AaEcRLBD+F). The 3D model of the depicted structure of AaEcRLBD+F (green) was generated using the I-TASSER server for predicting the 3D structure of the protein (http://zhanglab.ccmb.med.umich.edu/I-TASSER/(accessed on 14 August 2020)) [87] using the sequence of the full-length AaEcR (GenBank: AAA87394.1). All structures were visualized using PyMOL (PyMOL Molecular Graphics System, Version 2.0.4, Schrödinger, LLC, New York, NY, USA).

*A. aegypti* mosquitoes have been the main vectors of the world’s most devastating human diseases in recent years, such as dengue, Zika, yellow fever and chikungunya [82,83,84,85]. Many crucial processes in mosquitos’ reproduction follow the formation of an active heterodimeric complex of the 20E receptor (EcR) and *Ultraspiracle* protein (Usp) [86]. The EcR of *A. aegypti* (AaEcR) contains one of the longest F domains in known members of NRs (Figure 2), and it clearly stands out structurally from the evolutionary conserved overall canonical folds of NR LBDs and their C-terminal extensions (Figure 3).

It was previously shown that recombinant AaFEcR is mainly disordered with residual ordered secondary structures and takes the conformation of a premolten globule (PMG) [11]. It can undergo both induced folding (in the presence of trifluoroethanol) and unfolding (in the presence of guanidinium chloride). The increased content of His and Pro residues (compared to SwissProt database [11]) is not accidental, as it strongly resembles the amino acid motif present in His-Pro-rich glycoproteins (HPRGs) that is known to bind Zn^2+^ and Cu^2+^ ions [88]. Mass spectrometry results clearly showed the formation of AaFEcR-Zn^2+^ and AaFEcR-Cu^2+^ complexes and their stoichiometry. Coordination of Cu^2+^ and Zn^2+^ ions to this domain did not induce any statistically relevant changes in its overall secondary structure [11]. Further studies were conducted on two model peptides (Ac-HGPHPHPHG-NH_2_ and Ac-QQLTPNQQQHQQQHSQLQQVHANG-NH_2_) contained in the sequence of AaFEcR. The most fascinating result was observed for the interaction between the Ac-HGPHPHPHG-NH_2_ peptide and Cu^2+^ ions, which resulted in the formation of the rare and unique polyproline type II helical structure (PPII) [10]. Our findings were the first to show the Cu^2+^ binding-induced formation of PPII—a structure, which had not been reported for any NR superfamily member before. PPII helixes responsible for protein-protein and protein-DNA interactions [89,90,91,92]. However, the ion binding-dependent functional potential of AaFEcR remains to be solved.

### 4.2. AaFEcR Provokes LLPS

**Figure 4:**
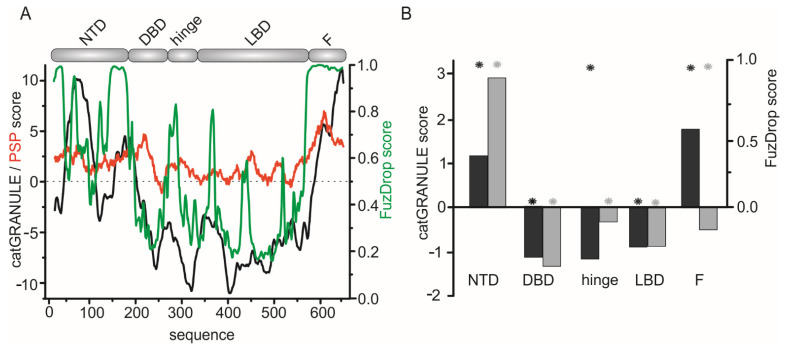
In silico analyses of the phase separation propensity of AaEcR. (**A**) Analysis performed for the sequence of EcR from *A. aegypti*. The propensity profiles refer to a scheme depicting the modular structure of the protein (above). Values above 0 indicate a putative tendency for provoking LLPS predicted by catGRANULE (black) and PSP (red) predictors, whereas values above 0.5 indicate a probable tendency predicted by FuzDrop (green). (**B**) Averaged propensity score values obtained for fragments of the EcR from *A. aegypti* (GenBank: AAA87394.1), and *D. melanogaster* (Uniprot: P34021). The black (*A. aegypti*) and grey (*D. melanogaster*) bars show the analysis performed using the catGRANULE predictor, asterisks show the results of an analysis performed using FuzDrop. The used predictors are available at: catGRANULE: http://s.tartaglialab.com/update_submission/329388/68a2757da8 (accessed on 15 September 2020); PSP: http://abragam.med.utoronto.ca/~JFKlab/Software/psp.htm (accessed on 15 September 2020); FuzDrop: http://protdyn-fuzpred.org/ (accessed on 26 January 2021).

The EcR is an important transcription regulator in arthropods. The physiological function of AaFEcR at the molecular level, especially in the activities of the full-length AaEcR, is still enigmatic. To investigate if the functionality of AaEcR may depend on the propensity of the AaFEcR formation of liquid condensates, bioinformatic analysis was initially performed. In the case of AaEcR, in silico sequence analysis performed using catGRANULE, PSP [93] and FuzDrop [94] predictors indicates that certain fragments may provoke LLPS especially in the N- and C-terminal fragments (Figure 4A). Comparative analysis of the EcR sequences between the receptor sequences from *A. aegypti* and *D. melanogaster*, which possess one of the longest F domains, indicates that both N-terminal domains can probably lead to the formation of liquid condensates (Figure 4B). In both cases, central fragments of the proteins containing folded DBDs and LBDs are characterized by negative scores, which suggests that these protein fragments may lack the ability for LLPS. The predictions obtained for the hinge and F regions gave interesting results. For the hinge region of EcR from *D. melanogaster*, catGRANULE and FuzDrop predict no propensity for provoking LLPS. For AaFEcR the analysis gave opposite results, i.e., a strong tendency for LLPS was predicted by FuzDrop and a lack of tendency was predicted by catGRANULE. For the sequence related to AaFEcR, high positive scores were obtained in the case of both predictors, whereas for the region of *D. melanogaster* the analysis was also ambiguous and inconclusive. As stated before, the F regions are the most variable fragments of NRs and they may have remarkable different molecular properties.

**Figure 5:**
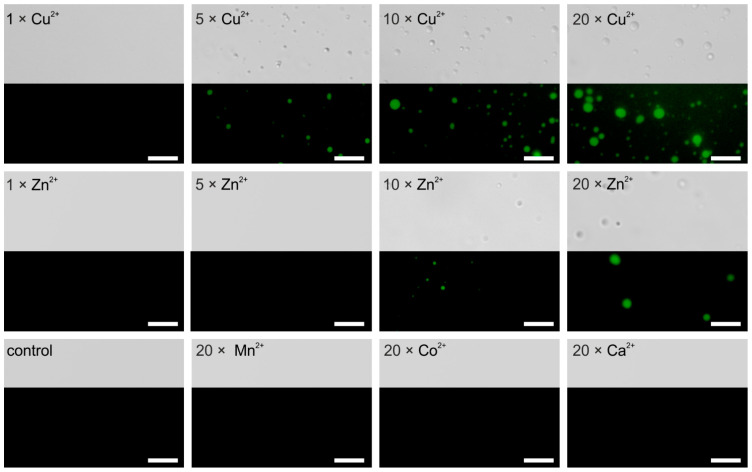
Metal ions-induced formation of liquid condensates by AaFEcR. The representative images of condensates observed using widefield microscopy with Differential Interference Contrast (DIC) (top) and fluorescence (bottom). The 70 µM solution of fluorescently labelled recombinant AaFEcR in 10 mM Tris-HCl, 150 mM NaCl, and pH 7.5 buffer was analyzed. The protein solution was supplemented with various metal ions in molar excess as indicated on the image. Scale bar 5 µm.

For further examination, in vitro analysis of LLPS using the isolated AaFEcR domain was performed. The fluorescently labeled domain was examined microscopically in different conditions. At first, we performed a series of experiments to test if the domain could form condensates in a ligand-independent manner. We tested different concentrations of the protein (ranging from 10–230 µM) in a Tris buffer containing 150 mM sodium chloride; next we tested the F domain (70 µM) in a buffer containing different sodium chloride concentrations ranging from 150–700 mM. Finally, the protein at 70 µM was analyzed in the presence of various buffer additives such as glycerol, polyethylene glycol 300 and 8000, ficol, hexanediol (for more details refer to Materials and Methods section in the Supplementary Materials). The formation of liquid condensates was not observed in any of the tested samples (data not shown). Inspired by our previous studies [10,11], the metal ions were included in the analyses. We found here that for the protein analyzed at 70 µM (1 mg/mL), 5–20× molar excess of Cu^2+^ and 10–20× molar excess of Zn^2+^ ions can induce the formation of condensates (Figure 5). Obtained results indicate that Cu^2+^ ions has a stronger effect since the ions can provoke the formation of condensates at lower molecular excess, whereas the effect of Zn^2+^ ions is weaker since its concentration to provoke phase separation needs to be higher. Other tested metal ions such as Co^2+^, Ca^2+^, and Mn^2+^ have no effect and, even in a larger molecular excess (20×), the solution remains in a one-phase regime (Figure 5).

**Figure 6:**
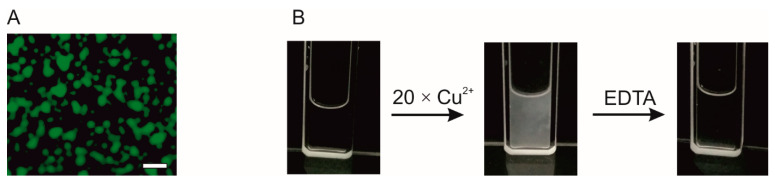
AaFEcR domain containing condensates exhibit liquid properties. (**A**) shows condensates formed by 70 µM fluorescently labeled F domain in 10 mM Tris-HCl, 150 mM NaCl, and pH 7.5 buffer supplemented with 20× molar excess of Cu^2+^ ions. The condensates wet the glass slide. (**B**) The changes of the F domain solution turbidity caused by the addition of 20× molar excess of Cu^2+^ ions and EDTA. Scale bar 5 µm.

The condensates formed by the F domain in the presence of Cu^2+^ ions are spherical and dynamic (see Video S1 in the Supplementary Materials). Several minutes after formation, they settle on the glass slide and can fuse together forming larger droplets (Figure 6A). That behavior indicates that they have a liquid nature [95]. To further examine the type of the phase transition, we performed an experiment in which to the sample containing F domain in the presence of 20× molar excess of Cu^2+^ ions, EDTA was added. The addition of the chelating agent to divalent ions dissolved the condensates. The absorbance measured at 340 nm for the unlabeled F domain, 70 µM and 20× Cu^2+^ ions increased to 0.342. When EDTA was added to the sample containing protein condensates, the absorbance dropped to 0.020 and the solution became clear (Figure 6B). That observation indicates that the formation of the condensates is reversible. Taking into account the spherical shape of the condensates, the fact that after several minutes of their appearance in solution they wetted the glass slide and the fact that their formation is reversible, we conclude that the condensates possess a liquid nature and are formed via the LLPS.

In our previous paper [10], it was shown that Cu^2+^ ions induce the formation of the PPII helix in the AaFEcR domain. The presented observation indicates that this type of secondary structure can probably accompany LLPS.

Presently, the knowledge regarding metal ions-induced LLPS is scattered [96,97] and this is one of the first reports of the Zn^2+^ and Cu^2+^-induced LLPS of disordered proteins. Nonetheless, the putative role of the Cu^2+^-induced PPII in these processes at the molecular level is waiting to be revealed. At present, however, we know little about the physiological consequences of the formation of metal-induced liquid condensates via LLPS by the F domain. Ion binding by AaFEcR, leading to the formation of liquid condensates, may enhance the assembly of AaEcR transcriptional complexes. AaFEcR may serve as a scaffold for metal ion-gated liquid condensates and may also allow various client molecules to come into the condensate, in turn contributing to the formation of a complete transcriptional machinery. It is also possible that metal ion-induced LLPS of AaFEcR leads to changes in the specificity or affinity of the interaction of the full-length AaEcR with DNA, appropriate ligands, or the receptor’s partners [10,11]. The physiological levels of Cu^2+^ and Zn^2+^ ions required to introduce the effector functions in EcR are unknown. In addition, not fully understood are the changes in mosquito cells’ homeostasis upon infection. However, it can be expected that the presence of pathogens may alter to some extent mosquito cells’ physiology and infection may trigger some stress-responsive mechanisms. Stress-induced changes of cell physiology reflect a changed pattern of gene expression [98]. The relation between the pathogen infection and metal ions-induced formation of liquid condensates is currently a puzzle. Yet, understanding of this phenomenon would enable a deeper insight into the mechanism that controls the life cycle and the basic developmental processes of *A. aegypti*.

## 5. Summary and Perspectives on Future Research

Mosquito transmitted diseases stand out for their universality and scale, but they have still not yet been overcome. There is no successful vaccine against Zika virus. The vaccine against all 4 serotypes of the dengue virus (*Dengvaxia*), which was approved by the FDA in 2019, is not recommended for people who have not been infected with the virus in the past [99]. The RTS,S malaria vaccine only provides partial protection [100,101]. Considering vaccine imperfection and the fact that the drug treatment of diseases is not 100% effective, it is essential to find other ways to protect exposed people. Researchers should either focus on reducing the mosquito population, or try to strengthen the immune reaction of people to mosquito-borne viruses or parasite invasion. The reduction of the mosquito population can be influenced by the EcR signaling pathway. By a deep understanding of the LLPS formation by its IDRs, we could change its transcriptional machinery gathering pattern. It is assumed that the multivalent macromolecular interactions are one of the necessary conditions for the formation of biomolecular condensates. In many cases, LLPS is driven by multivalent interactions between IDPs or IDRs and/or their protein or nucleic acid specific partners [102]. One can speculate that EcR from *A. aegypti* may use its intrinsically disordered F domain with the Cu^2+^-induced PPII helical structure as the platform for multiple interactions with modulatory proteins controlling gene expression under the control of specific ligands. By modulating the availability of metal ions, the LLPS formation could be disrupted in the appropriate signaling pathway. The 20-hydroxyecdysone signaling is strongly believed to be a promising target for the chemical control of malaria vectors [103]. It also regulates the physiological processes associated with vector competence and the abundance of mosquito vectors [103]. Thus, in a controlled manner, we could vastly and safely reduce the mosquito population and reduce the number of dengue, Zika fever and malaria infections. Undoubtedly, transcription factors play an important role in both the reproduction of mosquitoes and the immune response to Zika, dengue and *P. falciparum* inflammation. Various pathways are under their control, and therefore transcription factors, together with NRs, can be treated as an attractive target to strengthen an organism’s immune response. Deepening the knowledge about each element of the NRs’ structure will reveal the detailed mechanism of their action. A significant part of previous studies of the NRs’ structure was conducted using in vitro methods and recombinant proteins. Single-molecule in vivo studies of full-length NRs, supported by the recent technological advances in live-cell microscopy, should be applied to this new area of science. We believe that detailed studies of the mechanisms of LLPS in the activities of NRs during transcriptional events will significantly contribute to the development of new strategies to combat not only mosquito-transmitted diseases, but also cancer and other pathological disorders.

## Data Availability

Data available within the article and its Supplementary Materials.

## Supplementary Materials

The following are available online at https://www.mdpi.com/2073-4409/10/3/571/s1, Materials and Methods, Figure S1: The F domains of the insects’ ecdysone receptors vary in sequence and length, Figure S2: The F domains of the representative members of the mammalian nuclear receptors and AaFEcR vary in sequence and length, Figure S3: In silico analyses of disorder occurrence in the F domains of the representative nuclear receptors, Video S1: The condensates formed by the F domain in the presence of Cu^2+^ ions.
