# Supplementary material for: Metal Ions Induce Liquid Condensate Formation by the F Domain of Aedes aegypti Ecdysteroid Receptor. New Perspectives of Nuclear Receptor Studies
Source: Cells. 2021 Mar 5;10(3):571. doi: 10.3390/cells10030571 (PMC7999165; doi:10.3390/cells10030571)
Supplement: Supplementary file 1 [file cells-10-00571-s001.zip › cells-1053628-suppl proof/cells-1053628-proof suppl.pdf]

cells MDPI

Supplementary Materials

1. Materials and Methods

1.1. Structural modeling of AaEcRLBD with the F domain (AaEcRLBD+F)

Presented in Figure 3 the 3D model of AaEcRLBD+F covers the sequence of AaEcR from P331 to V675. The threading templates used by I-TASSER were as follows (according to the PDB library and listed from the top to bottom): 3e00D, 4nqaA, 4nqaB, 4nqa and 5uanB. After performing the structure assembly simulation, I-TASSER used the TM-align structural alignment program to match the first I-TASSER model to all structures in the PDB library. Proteins structurally very close to the target (AaEcR) or to its fragments in the PDB library (the highest TM-score to the predicted I-TASSER model) were found as follows (according to the PDB library and listed from the top to bottom): 4nqaB, 2aclH, 3l0eA, 3e00D, 3a40X, 6ez8A, 5m5zA, 5w1hA, 3eqnA and 2nxxE. The resulting predicted 3D model of the full-length AaEcR was assessed according to a confidence score (C-score). According to the score description in the I-TASSER results, the C-score is typically in the range of [−5, 2]. The 4nqaB structure was considered by the algorithm reliable (TM-score = 0.405, RMSD = 4.47, IDEN = 0.327, and Cov = 0.493) and used for the data interpretation. The best full-length AaEcR TOP5 model possessed C = −3.57 (estimated TM-score = 0.32 ± 0.11). Here, only the 3D model of the AaEcRLBD+F is shown from this structure. The presented 3D model of the F domain also fits well with the recently obtained experimental data [1,2].

1.2. Preparation of the Fluorescently Labeled AaFEcR

The recombinant F domain from *A. aegypti* was expressed and purified as described in previous work [1]. For fluorescence microscopy, the protein was labelled with Atto 488 NHS ester (Sigma-Aldrich). The purified domain was first transferred into the labeling buffer using the Superdex 75 Increase 10/300 G L (GE Healthcare) column. The buffer was prepared by mixing PBS buffer (with a pH of 7.4) and 0.2 M sodium bicarbonate solution (with a pH of 9) in a 20:1 ratio. The pH of the labeling buffer was 8.3. The fractions eluted from the column were pooled and concentrated to 2 mg/ml using the Amicon Ultra-4 Centrifugal Filter Unit (Merck/Millipore; molecular weight cut-off 10.0 kDa). Subsequently, the two-fold molar excess of the Atto 488 (Sigma-Aldrich) dye was added to the concentrated protein solution. The conjugation reaction was carried out at 22 °C and at 400 rpm for 1 h. To separate the unbound fluorescence dye from the protein, the reaction mixture was loaded onto the Superdex 75 Increase 10/300 GL (GE Healthcare) column equilibrated with 10 mM Tris-HCl buffer containing 150 mM NaCl (pH 7.5).

1.3. Phase Separation Assay

For microscopic examination, the labeled F domain (in 10 mM Tris-HCl, 150 mM NaCl, and pH 7.5) was concentrated to 3 mg/ml (227 μM) using the Amicon Ultra-4 Centrifugal Filter Unit (Merck/Millipore; molecular weight cut-off 10.0 kDa). That concentrated solution was next used to prepare other working solutions. At first, the F domain was analysed without additives at various concentrations ranging from 0.13 - 3 mg/ml. Next, the protein at 1 mg/ml (70 μM) was analyzed in a Tris buffer containing different concentration of sodium chloride ranging from 150 – 450 mM. The LLPS propensity of the F domain was also analyzed in the presence of different buffer additives. The protein at 70 μM concentration was analysed in presence of 10% glycerol, 50 and 25% of polyethylene glycol 3000 and 8000, ficol, and 10% of hexanediol. The analysed additives were first dissolved in 10 mM Tris-HCl, 150 mM NaCl, pH 7.5 and next the solutions were mixed with the labelled F domain to obtain the target concentration of the protein and additives. The samples containing different metal ions in the form of MCl_2 (M refers to Cu^{2+}, Zn^{2+}, Co^{2+}, Mn^{2+}, and Ca^{2+}) were prepared analogically. Prior microscopic examination, all prepared solutions containing the labeled F domain were incubated for 2 min at room temperature. For the observations, Axio Observer 7 (Carl Zeiss) inverted microscope with the 100 × oil and 1.3 numerical aperture objective lens was used. The widefield differential interference contrast (DIC) and fluorescence images were collected with the Axiocam 305 color camera (Carl Zeiss).

For the determination of the reversibility of the phase transition, the sample containing 70 μM of the unlabelled F domain and 20× molar excess of Cu^{2+} ions was prepared by mixing the appropriate volumes of their stock solutions. 5 min after mixing, to 400 µl of the tested solution, 50 μL of the 100 mM solution of EDTA in the Tris buffer was added. These samples were analyzed spectroscopically by measuring the absorbance at 340 nm using NanoDrop spectrophotometer (Thermo Scientific).

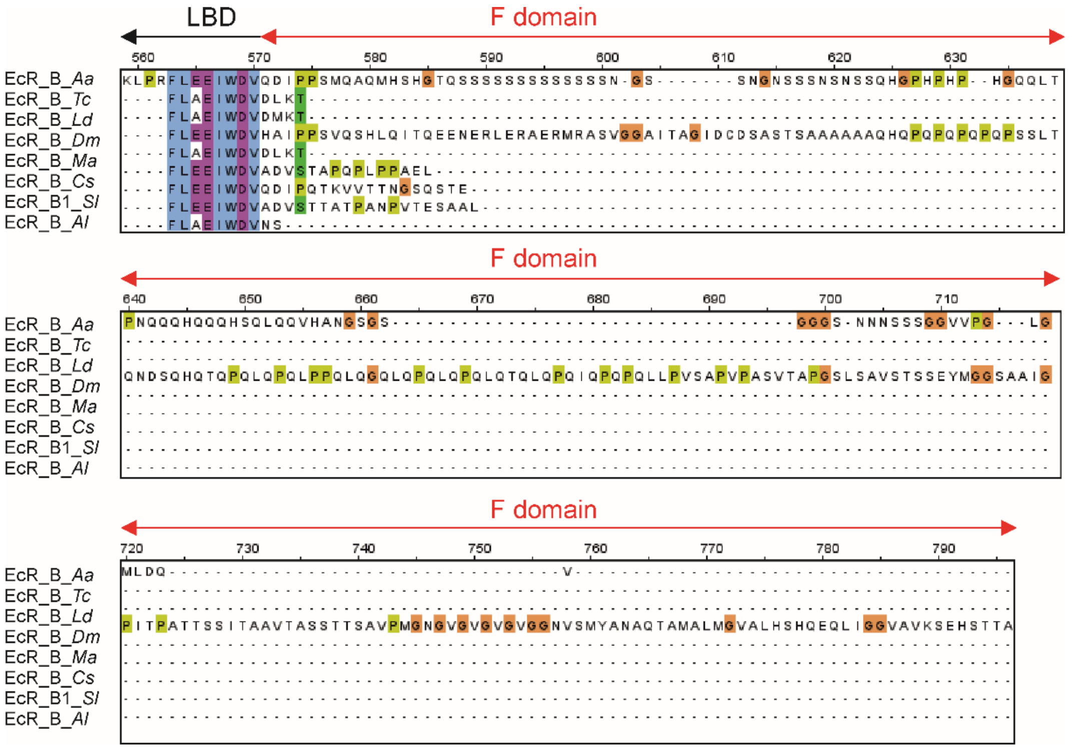

Figure S1. The F domains of the insects' ecdysone receptors vary in sequence and length. The used abbreviations and the accession numbers (in parentheses) regard isoforms B of some insects' ecdysone receptors. The amino acid sequences following the last helix of EcR_B_LBD were compared and their references are as follows: *Aedes aegypti* (EcR_B_Aa, GenBank: AAA87394.1), *Tribolium castaneum* (EcR_B_Tc, NCBI: NP_001135390.1), *Leptinotarsa decemlineata* (EcR_B_Ld, GenBank: QBH70334.1), *Drosophila melanogaster* (EcR_B_Dm, NCBI: NP_724460.1), *Monochamus alternates* (EcR_B_Ma, GenBank: AEY63781.1), *Conopomorpha sinensis* (EcR_B_Cs, GenBank: QOU81377.1), *Spodoptera litura* (EcR_B1_Sl, GenBank: AFK27931.1), *Apolygus lucorum* (EcR_B_Al, GenBank: AIZ50655.1). Residue numbering corresponds to the sequence of EcR_B_Aa (GenBank: AAA87394.1). All representative amino acid sequences were compared using the ClustalΩ tool [3] and visualized in Jalview [4].

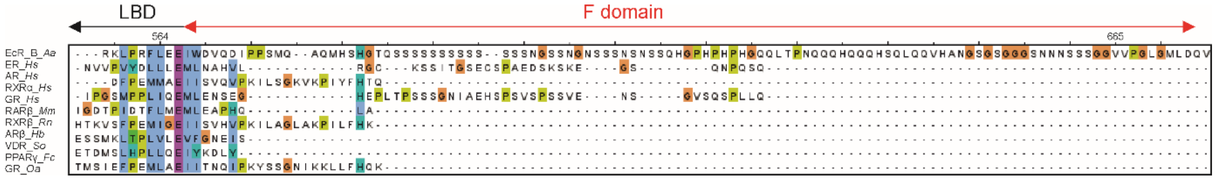

Figure S2. The F domains of the representative members of the mammalian nuclear receptors and AaFEcR vary in sequence and length.

The used abbreviations and the accession numbers (in parentheses) regard some mammal nuclear receptors compared to ecdysteroid receptor isoform B from *A. aegypti* (EcR_B_*Aa*, GenBank: AAA87394.1). The amino acid sequences following the last helix of LBD were compared and their references are as follows: estrogen receptor from *Homo sapiens* (ER_*Hs*, UniProt: Q92731.2), androgen receptor from *H. sapiens* (AR_*Hs*, UniProt: P10275.3), retinoid X receptor α from *H. sapiens* (RXRα_*Hs*, UniProt: P19793.1), glucocortycoid receptor from *H. sapiens* (GR_*Hs*, UniProt: P04150.1), retinoic acid receptor β from *Mus musculus* (RARβ_*Mm*, UniProt: P22605.1), retinoid X receptor β from *Rattus norvegicus* (RXRβ, GenBank: CAE83933.1), androgen receptor β from *Haplochromis burtoni* (ARβ_*Hb*, GenBank: AAL92878.2), vitamin D receptor from *Saguinus Oedipus* (VDR_*So*, GenBank: AAK48863.1), peroxisome proliferator-activated receptor γ from *Felis catus* (PPARγ_*Fc*, NCBI: NP_001106647.1), glucocorticoid receptor from *Ovis aries* (GR_*Oa*, NCBI: NP_001107658.1). Residue numbering corresponds to the sequence of EcR_B_*Aa* (GenBank: AAA87394.1). All representative amino acid sequences were compared using the ClustalΩ tool [3] and visualized in Jalview [4].

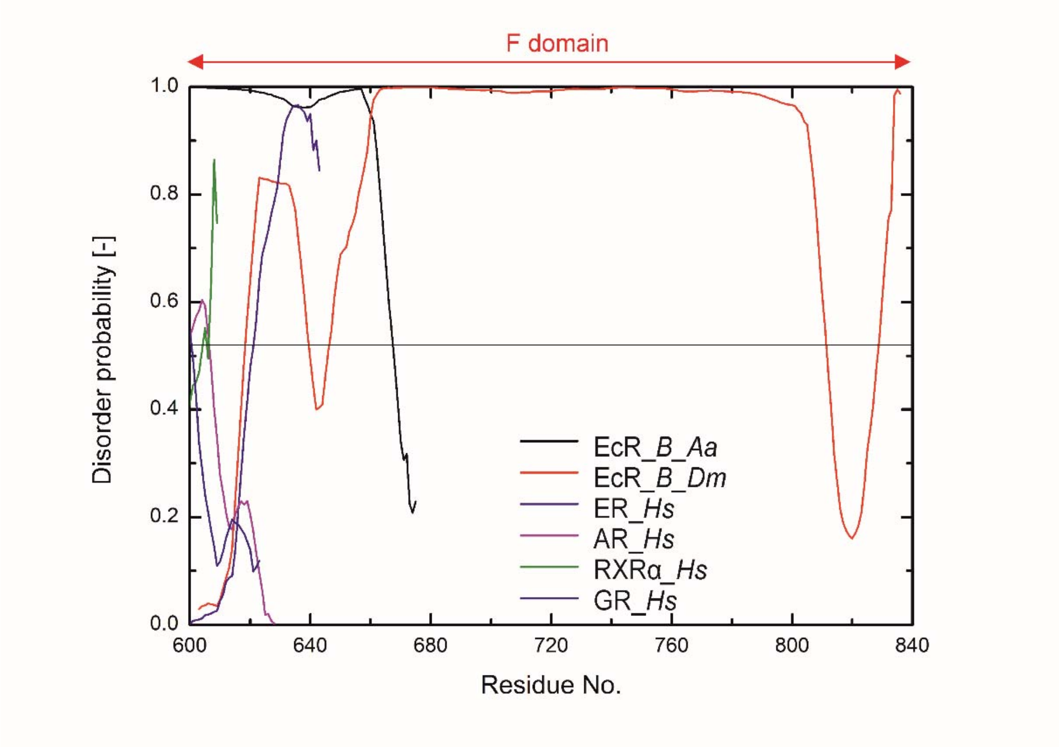

Figure 3. In silico analyses of disorder occurrence in the F domains of the representative nuclear receptors. Intrinsic disorder probability was examined with PONDR VLXT algorithm [5] for F domains of ecdysteroid receptor isoform B from *A. aegypti* (EcR_B_Aa, GenBank: AAA87394.1), ecdysone receptor isoform B from *D. melanogaster* (EcR_B_Dm, NCBI: NP_724460.1), estrogen receptor from *H. sapiens* (ER_Hs, UniProt: Q92731.2), androgen receptor from *H. sapiens* (AR_Hs, UniProt: P10275.3), retinoid X receptor α from *H. sapiens* (RXRα_Hs, UniProt: P19793.1), glucocortycoid receptor from *H. sapiens* (GR_Hs, UniProt: P04150.1). retinoic acid receptor β from *Mus musculus* (RARβ_Mm, UniProt: P22605.1). Residue numbering corresponds to the sequence of EcR_B_Aa (GenBank: AAA87394.1).

Video S1. The condensates formed by the F domain in the presence of Cu^{2+} ions.

References:

1. Więch, A.; Rowińska-Żyrek, M.; Wątły, J.; Czarnota, A.; Hołubowicz, R.; Szewczuk, Z.; Ożyhar, A.; Orłowski, M. The intrinsically disordered C-terminal F domain of the ecdysteroid receptor from Aedes aegypti exhibits metal ion-binding ability. *J. Steroid Biochem. Mol. Biol.* **2019**, *186*, 42–55, doi:10.1016/j.jsbmb.2018.09.008.
2. Rowińska-Żyrek, M.; Więch, A.; Wątły, J.; Wieczorek, R.; Witkowska, D.; Ożyhar, A.; Orłowski, M. Copper(II)-binding induces a unique polyproline type II helical structure within the ion-binding segment in the intrinsically disordered F-domain of ecdysteroid receptor from Aedes aegypti. *Inorg. Chem.* **2019**, *58*, 11782–11792, doi:10.1021/acs.inorgchem.9b01826.
3. Li, W.; Cowley, A.; Uludag, M.; Gur, T.; McWilliam, H.; Squizzato, S.; Park, Y.M.; Buso, N.; Lopez, R. The EMBL-EBI bioinformatics web and programmatic tools framework. *Nucleic Acids Res.* **2015**, *43*, W580-4, doi:10.1093/nar/gkv279.
4. Waterhouse, A.M.; Procter, J.B.; Martin, D.M.A.; Clamp, M.; Barton, G.J. Jalview Version 2--a multiple sequence alignment editor and analysis workbench. *Bioinformatics* **2009**, *25*, 1189–1191, doi:10.1093/bioinformatics/btp033.
5. Peng, K.; Vucetic, S.; Radivojac, P.; Brown, C.J.; Dunker, A.K.; Obradovic, Z. Optimizing long intrinsic disorder predictors with protein evolutionary information. *J. Bioinform. Comput. Biol.* **2005**, *3*, 35–60.
