# Supplementary figures and images for: Metal Ions Induce Liquid Condensate Formation by the F Domain of Aedes aegypti Ecdysteroid Receptor. New Perspectives of Nuclear Receptor Studies
Source: Cells. 2021 Mar 5;10(3):571. doi: 10.3390/cells10030571 (PMC7999165; doi:10.3390/cells10030571)

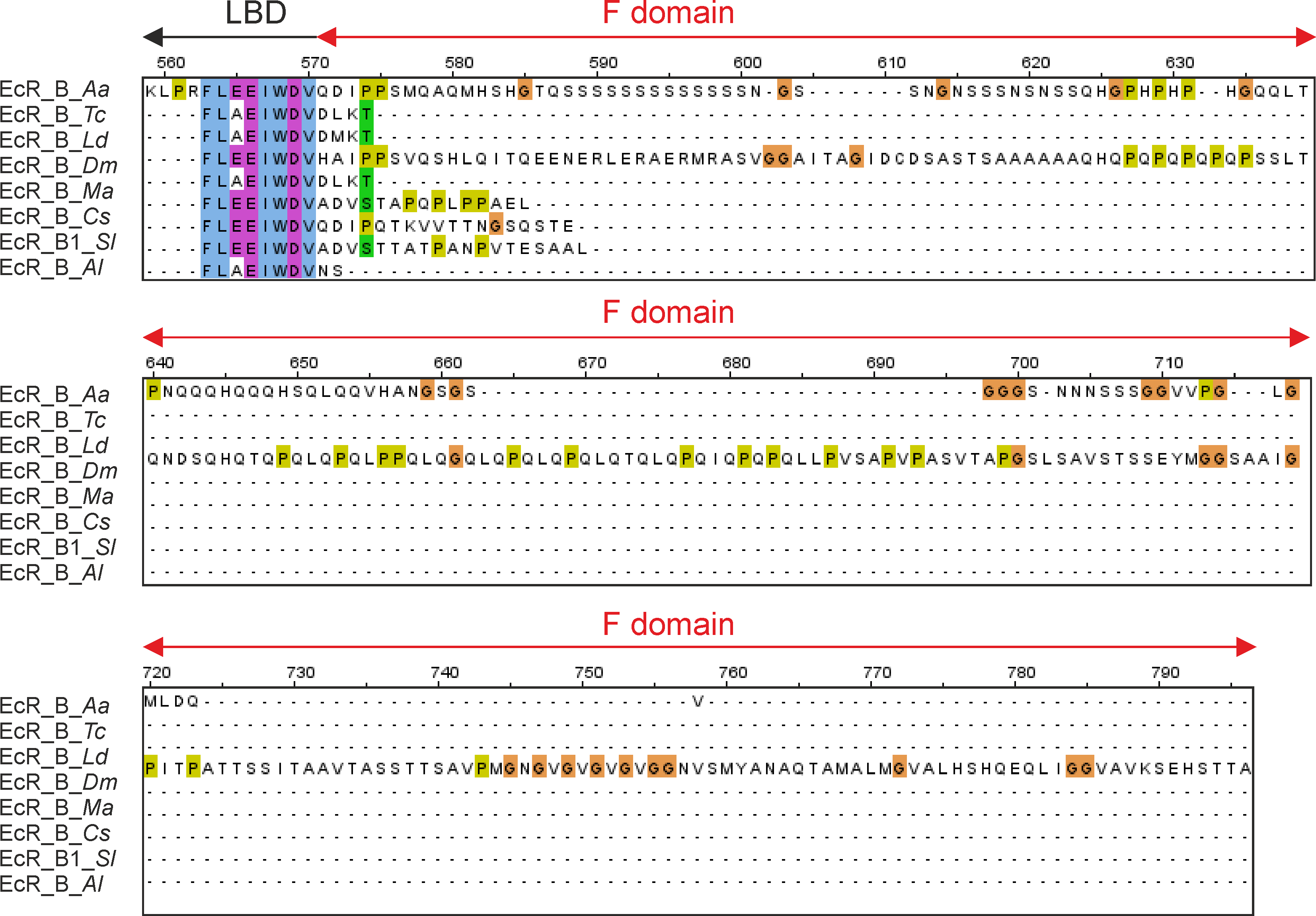

Supplement: Supplementary file 1 [file cells-10-00571-s001.zip › cells-1053628-suppl proof/Figure S1.tif]

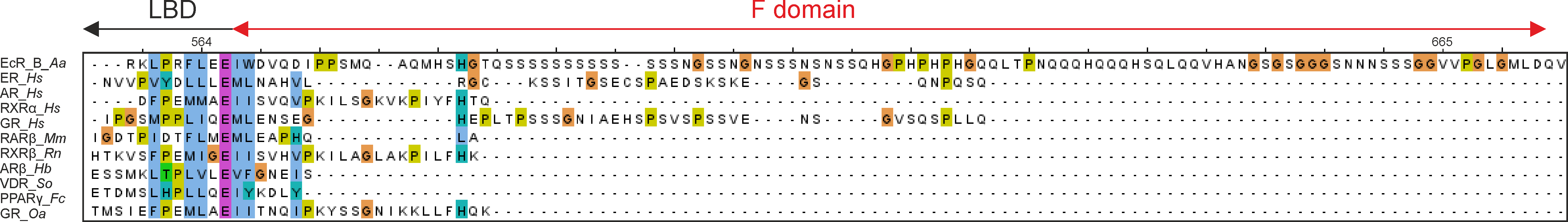

Supplement: Supplementary file 1 [file cells-10-00571-s001.zip › cells-1053628-suppl proof/Figure S2.tif]

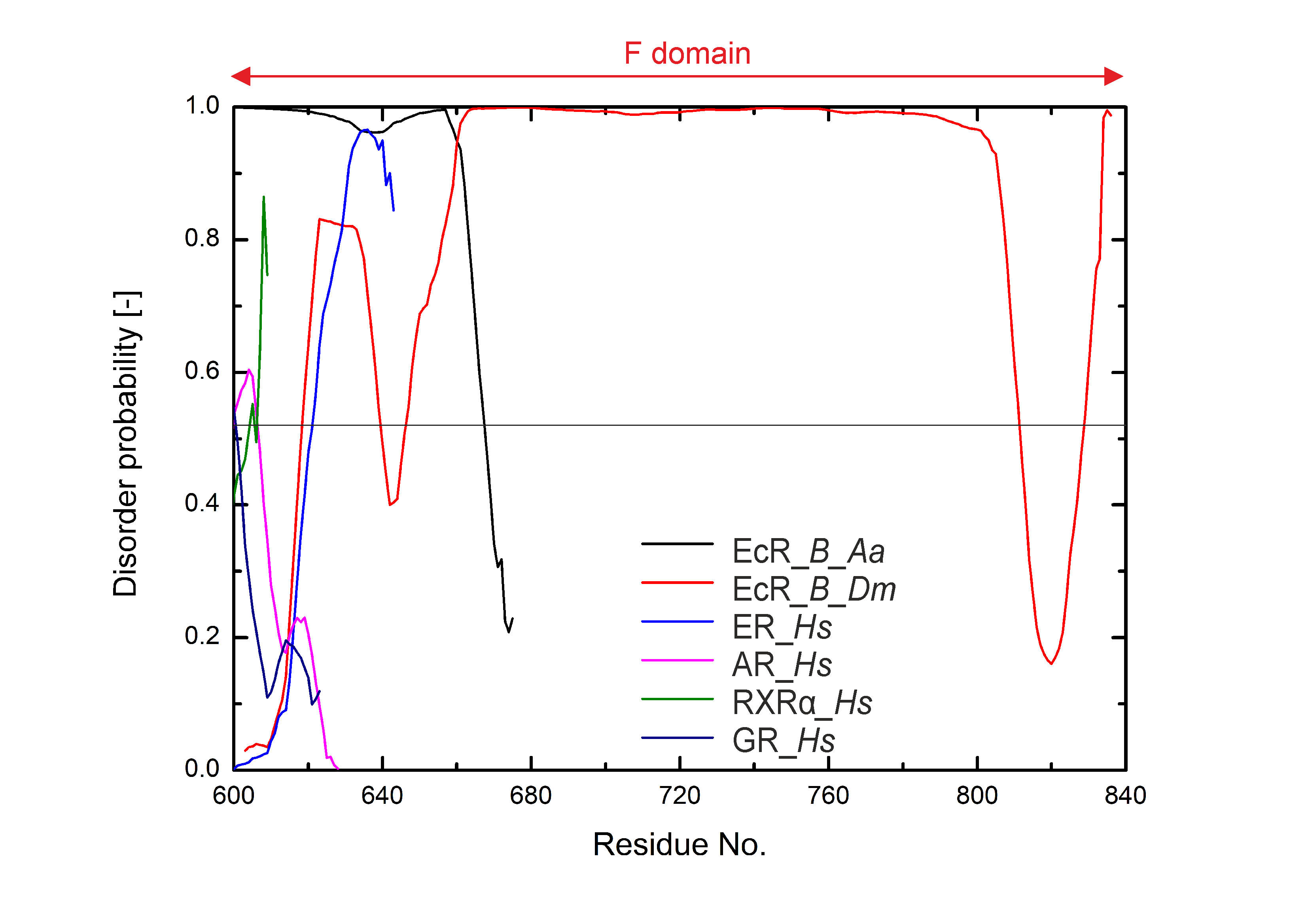

Supplement: Supplementary file 1 [file cells-10-00571-s001.zip › cells-1053628-suppl proof/Figure S3.tif]
